# Variegated Squirrel Bornavirus 1 in Squirrels, Germany and the Netherlands

**DOI:** 10.3201/eid2303.161061

**Published:** 2017-03

**Authors:** Kore Schlottau, Maria Jenckel, Judith van den Brand, Christine Fast, Christiane Herden, Dirk Höper, Timo Homeier-Bachmann, Jens Thielebein, Niels Mensing, Bert Diender, Donata Hoffmann, Rainer G. Ulrich, Thomas C. Mettenleiter, Marion Koopmans, Dennis Tappe, Jonas Schmidt-Chanasit, Chantal B.E.M. Reusken, Martin Beer, Bernd Hoffmann

**Affiliations:** Friedrich-Loeffler-Institut, Greifswald-Insel Riems, Germany (K. Schlottau, M. Jenckel, C. Fast, D. Höper, T. Homeier-Bachmann, D. Hoffmann, R.G. Ulrich, T.C. Mettenleiter, M. Beer, B. Hoffmann);; Erasmus Medical Center, Rotterdam, the Netherlands (J. van den Brand, M. Koopmans, C.B.E.M. Reusken);; Justus-Liebig University Gießen, Gießen, Germany (C. Herden);; Martin Luther University Halle-Wittenberg, Halle, Germany (J. Thielebein);; Tierarztpraxis Dr. Niels Mensing, Magdeburg, Germany (N. Mensing);; Dierenartspraktijk Diender, Vlissingen, the Netherlands (B. Diender);; Bernhard Nocht Institute for Tropical Medicine, Hamburg, Germany (D. Tappe, J. Schmidt-Chanasit)

**Keywords:** viruses, zoonoses, bornavirus, squirrels, variegated squirrel bornavirus 1, VSBV-1, Germany, the Netherlands

## Abstract

We screened squirrels in Germany and the Netherlands for the novel zoonotic variegated squirrel bornavirus 1 (VSBV-1). The detection of VSBV-1 in 11 squirrels indicates a considerable risk for transmission to humans handling those animals. Therefore, squirrels in contact with humans should routinely be tested for VSBV-1.

The family *Bornaviridae* comprises the classical mammalian bornaviruses (*Mammalian 1 bornavirus* with borna disease virus; BoDV-1 and -2); avian bornaviruses (*Passeriform 1/2 bornavirus*, *Psittaciform 1/2 bornavirus*, *Waterbird 1 bornavirus*); and a recently described *Elapid 1 bornavirus* from snakes (Loveridge’s garter snake virus 1) (*1*). BoDV-1 and -2 are considered nonzoonotic (*6*–*8*). The bicolored white-toothed shrew (*Crocidura leucodon*) has been proposed as a reservoir species for BoDV-1 (*2*–*5*). In addition to the exogenous viruses, endogenous bornavirus-like genomic elements have been found within the genome of humans and several animal species, including the 13-lined ground squirrel (*Spermophilus tridecemlineatus*) (*9*,*10*).

In 2015, a novel zoonotic bornavirus, the variegated squirrel bornavirus 1 (VSBV-1; new species *Mammalian 2 bornavirus*), was discovered in tissue samples from the central nervous system (CNS) of 3 persons with encephalitis, which resulted in death (*11*). The patients were breeders of variegated squirrels (*Sciurus variegatoides*), and an almost identical bornavirus sequence was detected in 1 contact squirrel kept by one of the breeders. Phylogenetic analysis classified VSBV-1 as a unique member of a new species of the genus *Bornavirus* (*11*).

We identified more VSBV-1 infected squirrels in Germany and the Netherlands. We also describe reliable molecular and serologic methods for in vivo detection of zoonotic VSBV-1 and its phylogenetic characterization.

## The Study

During 2015, samples were collected from 468 squirrels representing 14 species. Sampled squirrels were from varying locations: private holdings with up to 45 animals per holding, zoological gardens, or roadkill (Table 1). Locations represented 3 countries: Germany (399 samples from 28 holdings), the Netherlands (49 samples from 4 holdings), and the United Kingdom (20 samples from roadkill). Most live squirrels were sampled at the request of the owners. At least 1 swab sample and, if possible, duplicates of oral swab samples were collected from living animals, and brain samples were tested from dead squirrels. Blood samples (EDTA/heparin) were also available from 164 squirrels (Table 1). We obtained congruent results when we tested swabs and brain samples by 2 different VSBV-1–specific quantitative reverse transcription PCR (qRT-PCR) assays (*11*). Blood samples were centrifuged, and the resulting plasma was tested for the presence of viral RNA by using qRT-PCR and for bornavirus-specific IgG by using an indirect immunofluorescence assay (*11*).

**Table 1:** Results of testing for VSBV- in oral swab, blood, and brain samples from squirrels of 4 Sciuridae subfamilies collected in Germany, the Netherlands, and the United Kingdom, 2015*

Subfamily/species	No. positive/no. sampled	Available sample type					
		SS, B, BM	SS, BM	SS, B	BM, B	SS	BM
Callosciurinae							
* Callosciurus erythraeus*	0/7	0	4	2	1	0	0
* Callosciurus prevostii*	5/57	5	0	26	11	14	1
* Tamiops mcclellandii*	0/2	0	0	2	0	0	0
* Tamiops swinhoei*	0/4	0	0	0	0	4	0
Ratufinae							
* Ratufa macroura*	0/2	0	1	0	0	1	0
Sciurinae							
* Sciurus aureogaster*	0/5	0	0	0	0	5	0
* Sciurus carolinensis*	0/12	0	0	0	0	0	12
* Sciurus granatensis*	0/22	0	0	4	0	18	0
* Sciurus niger*	0/2	0	0	1	0	1	0
* Sciurus variegatoides*	7†/212	46	0	63	0	103	0
* Sciurus vulgaris*	0/127	0	2	1	0	62	62
Xerinae							
* Sciurotamias davidianus*	0/9	0	1	2	0	6	0
* Tamias sibiricus*	0/1	0	0	0	0	1	0
* Tamias striatus*	0/6	0	1	0	0	5	0
Total	12†/468	51	9	101	12	220	75

^*^* *Swab samples were taken from the oral cavity of each investigated animal. Oral swab samples and, if available, brain samples were tested by RT-qPCR. Blood samples (in EDTA or heparin), wherever available, were tested by quantitative reverse transcription PCR as well as by indirect immunofluorescence assay. B, blood, BM, brain material; SS, swab sample; VSBV-1, variegated squirrel bornavirus 1. †Includes the variegated squirrel in which VSBV-1 was initially discovered (*11*).

We detected VSBV-1 RNA and bornavirus-specific antibodies in 11 (2.6%) of the 468 animals. These 11 VSBV-1–positive squirrels (7 male, 4 female) belonged to the family Sciuridae, subfamilies Sciurinae (variegated squirrels, *S. variegatoides*; 6 animals) or Callosciurinae (Prevost’s squirrel, *C. prevostii*; 5 animals) (Table 1). None of the animals showed clinical signs associated with CNS disorders. Of the 11 VSBV-1–positive animals, 9 were from 4 squirrel breeders in Germany (holdings I–IV). The remaining 2 were from a private holding in the Netherlands (holding V). Of the 6 variegated squirrels, 3 originated from holding I, where the first VSBV-1–positive animal was identified in 2014 (*11*), and the remaining 3 were from holdings II and III. Squirrels from the 3 holdings were traded for breeding. Of the 5 VSBV-1–positive Prevost’s squirrels, 3 came from holding IV in Germany, and 2 were from holding V. The positive squirrels in the Netherlands had been transferred from a breeder in Germany in 2011–2012, but we could establish no direct epidemiologic link to the holdings in Germany where we found VSBV-1–positive animals.

**Figure 1:**
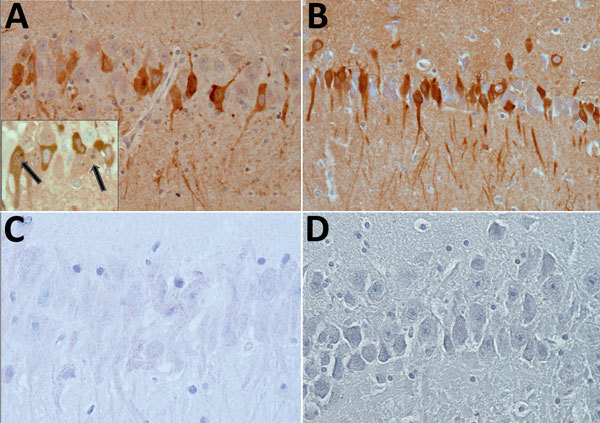
Immunohistochemical detection of bornavirus X protein (A) and phosphoprotein (B) in hippocampal neurons of a brain of a Prevost’s squirrel (*Callosciurus prevostii*) collected in Germany in 2015. Viral antigen is shown in nuclei or cytoplasm and processes. Inset shows intranuclear dot (inclusion body) in cells with and without cytoplasmic immunostaining (arrows). No staining was observed for bornavirus X protein (C) or phosphoprotein (D) in a bornavirus-negative variegated squirrel. Original magnification ×400

All animals with detectable virus as well as 40 negative squirrels from the same holdings were euthanized and checked for macroscopic lesions. We applied hematoxylin and eosin staining to formalin-fixed, paraffin-embedded brain sections from 9 of 11 animals and performed immunohistochemistry as described previously (*12*). A BoDV-positive horse brain sample served as positive control. Histopathology showed that 5 of 9 VSBV-1–positive animals, variegated squirrels as well as Prevost’s squirrels, had mild nonsuppurative meningitis or encephalitis. In 8 of 9 animals, we also detected intranuclear eosinophilic Joest-Degen inclusion bodies in scattered neurons of brain, spinal cord, and trigeminal as well as spinal ganglia (data not shown). We observed bornavirus-specific phosphoprotein and X protein throughout the brain in neurons, glial cells, and in a few ependymal cells in nuclei, cytoplasm, and cellular processes (Figure 1). We tested a panel of organ samples from all euthanized animals by qRT-PCR. All animals for which swab samples were VSBV-1–positive harbored considerable VSBV-1 genome loads, whereas animals for which swab samples were negative for viral RNA were also negative for viral loads in all tested organs (Table 2; data for control animals not shown). We found the highest viral RNA loads in the CNS and organs (kidney, nose, bladder, salivary gland, and sex organs), which could play a role in viral shedding and transmission (Table 2). Skin sections were also positive, indicating that VSBV-1 has broad cell and organ tropism. We were able to cocultivate infected primary squirrel cells with a permanent cell line and to isolate infectious virus from these passaged cells.

**Table 2:** VSBV-1 RNA levels in samples from squirrels collected in Germany and the Netherlands in 2015 that were positive for viral RNA and VSBV-1 antibodies*

Animal and sample type	Country and holding no.													
	Germany												The Netherlands	
	I	I		I	II	II	III	IV	IV		IV		V	V
Species	*Sciurus variegatoides*							*Callosciurus prevostii*						
Identity	48/15-1		48/15-2	48/15-11	49/15-1	49/15-6	75/15	122/15-1		122/15-2	133/15		3/16-1	3/16-2
Animal sex	M		F	M	M	F	M	F		M	M		F	M
EDTA blood/transudate	±		±	±	+	±	+	NA		NA	NA		±	±
Oral swab sample	+		±	++	+	+	+	+		+	+		NA	NA
Nose swab sample	±		±	+	+	±	+	+		±	±		NA	NA
Eye swab sample	+		±	+	+	±	+	NA		±	±		NA	NA
Palatine tonsil	++		++	++	++	+	++	++		++	++		+++	++
Mesenteric lymph nodes	++		++	+++	++	++	+	++		++	++		+	++
Brain	+++		+++	+++	+++	+++	+++	++		+++	+++		+++	+++
Brachial plexus	++		++	++	++	++	++	++		++	++		+	++
Medulla oblongata	+++		+++	+++	+++	+++	+++	++		++	++		+++	+++
Trigeminal ganglion	+		+	+++	+++	++	++	NA		++	++		++	+++
Kidney	++		+	++	+++	+++	++	++		++	++		++	+++
Salivary gland	++		+	+	++	++	++	++		++	++		±	+++
Urinary bladder	++		++	+++	+++	++	+	++		++	++		++	++
Lung	++		++	+++	+++	++	++	++		+	++		±	++
Heart	++		+	++	++	++	++	+		+	++		+	++
Spleen	+		±	++	++	+	++	++		++	++		±	+++
Liver	±		±	+	+	++	+	+		+	+		±	+
Pancreas	++		+++	++	++	++	++	++		++	++		+	++
Sex organ	+		+++	+	+++	+	+++	+++		++	++		++	+++
Skeletal muscles	+		±	+	++	+	++	++		++	++		+	++
Skin	++		+	++	++	++	++	++		++	++		+	++
Nose cross-section	++		++	+++	+++	++	+++	+		++	++		++	+++

*Data are represented in VSBV-1 genome equivalent copies per milliliter of template: +++, >10^6^; ++, 10^4^–10^6^; +, 10^2^–10^4^; ± 10^0^–10^2^). The VSBV-1–specific Mix 10 quantitative real- time PCR assay was used for quantification (limit of detection 1 genome equivalent/µL template). NA, sample not available; VSBV-1, variegated squirrel bornavirus 1.

We sequenced the VSBV-1 genome (8,786 nt; missing only the 5′ and 3′ noncoding regions) from all 11 squirrels and compared the sequences with the published VSBV-1 prototype sequence from a variegated squirrel (GenBank accession no. LN713680) (*11*). Accession numbers of the VSBV-1 sequences from this study are LT 594381–LT5943919 (European Nucleotide Archive).

**Figure 2:**
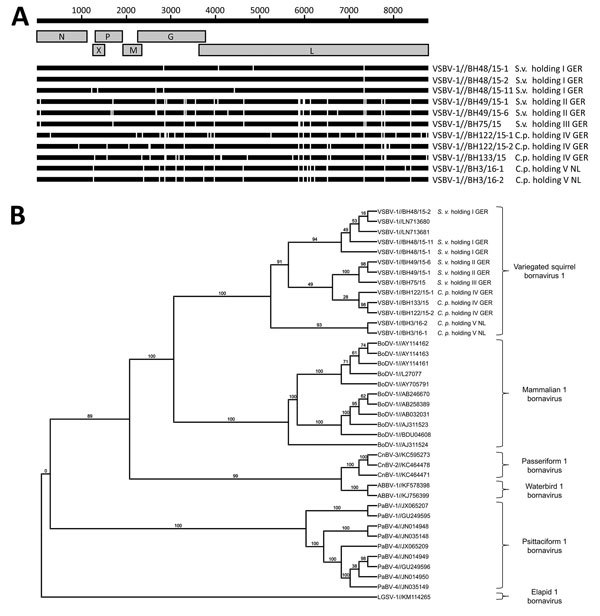
Analysis of 11 newly identified VSBV-1 genomes from squirrels collected in Germany and the Netherlands, 2015, in comparison with related bornaviruses. A) New sequences aligned with published squirrel-derived VSBV-1 genome (GenBank accession no. LN713680). The upper black bar indicates the reference sequence; gray boxes depict the genome. White bar sections for each animal sequence indicate nucleotide variations. The new sequences show 1–29 nucleotide differences compared to the published prototype sequence. G, glycoprotein; L, large structural protein; M, matrix; N, nucleoprotein; P, phosphoprotein; X, nonstructural protein. B) Phylogenetic tree of VSBV-1 isolates from this study (labeled) and comparison sequences. Tree was constructed using the maximum-likelihood method. Numbers along branches are bootstrap values. GER, Germany; NL, the Netherlands; VSBV-1, variegated squirrel bornavirus 1.

Genomes of viruses detected in squirrels from holding I showed the highest similarity with 1–6 nt substitutions, resulting in 1–3 aa changes, whereas the other sequences exhibited more variability. We found <29 nt substitutions causing <19 aa changes. The mutations were evenly distributed over the coding regions, but in viral genomes of squirrels from the same holding, they often occurred at the same position (Figure 2, panel A).

We aligned the 11 VSBV-1 sequences with those from other bornaviruses by using MAFFT (Multiple Alignment using Fast Fourier Transform), and we used the best-fit model TIM2+I+G4 to construct a phylogenetic tree (IQ-Tree version 1.3.10; http://iqtree.cibiv.univie.ac.at) with 1,000 bootstrap replicates. The novel VSBV-1 genomes clustered with the 2 human- and squirrel-derived prototype sequences LN 713680 and LN71368, forming a unique VSBV-1 clade separate from other mammal, avian, and snake bornaviruses (sequence identity <69%; Figure 2, panel B).

## Conclusions

We screened 468 squirrels and identified 11 VSBV-1–positive animals, including squirrels belonging to 2 subfamilies of the family Sciuridae. Although the VSBV-1–positive squirrels originated from different holdings and belonged to different subfamilies, the viral genome sequences formed a distinct VSBV-1 cluster. Whole-genome analyses provided no evidence for specific mutation patterns with regard to zoonotic potential or species-specific adaptations.

None of the VSBV-1-positive animals showed clinical signs of infection. Highest viral genome loads were found in the CNS, followed by the oral cavity and skin, indicating the potential for transmission to humans through scratching or biting. Only those squirrels positive for VSBV-1 by qRT-PCR displayed bornavirus-specific antibodies. Although serologic analyses support the qRT-PCR results, collecting serum samples is difficult and often not feasible for private breeders. Our data suggest that screening of swab samples is a suitable and reliable tool for noninvasive monitoring of squirrels for VSBV-1 infection. The prevalence of 3.3% for *S. variegatoides* and 8.8% for *C. prevostii* squirrels indicates a considerable risk for transmission to humans handling those animals without taking precautionary measures. We therefore recommend routine testing of squirrels in contact with humans, such as those in breeding and holding facilities or zoological gardens, at least from the subfamilies Sciurinae and Callosciurinae.
